# The remote effects of intravitreal anti-VEGF therapy

**Published:** 2016

**Authors:** F Balta, M Merticariu, C Taban, G Neculau, A Merticariu, D Muresanu, D Badescu, V Jinga

**Affiliations:** * Department of Vitreo-Retinal Surgery, Emergency Eye Hospital and Clinic, Bucharest, Romania,; ** Department of Urology, “Theodor Burghele” Clinical Hospital, Bucharest, Romania,; *** Romanian Centre for Economic Modeling,; **** RoNeuro” Institute for Neurological Research and Diagnostic, Cluj-Napoca, Romania,; ***** Department of Clinical Neurosciences, “Iuliu Hațieganu” University of Medicine and Pharmacy, Cluj-Napoca, Romania

**Keywords:** Avastin, benign prostatic hyperplasia, intravitreal

## Abstract

**Objective:** To study the effects of intravitreal anti-Vascular Endothelial Growth Factor (VEGF) therapy with Avastin for wet Age-Related Macular Degeneration (AMD) on Benign Prostatic Hyperplasia (BPH)-related symptoms.

**Methods:** An exploratory trial was conducted from August 1, 2013 to February 1, 2014, that included 14 male patients previously diagnosed with BPH, who were aged between 59 and 69 years. The trial was performed in Bucharest and involved two medical institutions: the Clinical Hospital of Eye Emergencies and the “Prof. Dr. Theodor Burghele” Hospital. This prospective study utilized both objective and subjective indicators to analyze the link between intravitreal anti-VEGF therapy for wet AMD and BPH. The evaluations consisted of uroflowmetry and International Prostate Symptom Score (I-PSS) assessments.

**Results:** The maximum flow rate (Qmax) improved by an average of 5.05 ml/ sec in 9 patients, whereas the remaining 5 patients showed a slight decrease in Qmax (mean 1.6 ml/ sec). The I-PSS score improved, with an overall decrease of 1.18 points at follow-up compared to the initial score (mean initial score = 2.42; mean follow-up score = 1.24).

**Conclusion:** The analysis revealed that anti-VEGF therapy for wet AMD had a significant positive effect on all BPH-related symptoms; patients reported improved urinary streams and decreased nocturia.

**Abbreviations:** BPH = benign prostatic hyperplasia, AMD = age-related macular degeneration, VEGF = vascular endothelial growth factor, I-PSS = international prostate symptom score, Qmax = maximum flow rate, TSP-1 = thrombospondin-1, FGF-2 = fibroblast growth factor, mRNA = precursor messenger ribonucleic acid, PSA = prostate-specific antigen, DRE = digital rectal examination, AUR = acute urinary retention, COX2 = cyclooxygenase 2, QoL = quality of life

## Introduction

Much research has focused on the key molecules that regulate new vessel formation. One of the most important angiogenic molecules is VEGF (Vascular Endothelial Growth Factor), also known as VPF (Vascular Permeability Factor), a potent and specific angiogenesis-related cytokine that is responsible for endothelial cell differentiation, migration, and proliferation as well as tubular formation and vessel assembly [**1**]. 

Recent reports in literature have addressed the importance of the VEGF system in the development of the normal prostate and prostatic hyperplasia. VEGF is one of the most potent regulators of angiogenesis and has been shown to act on two tyrosine kinase family receptors: c-fms-like tyrosine kinase (Flt-1) and fetal liver kinase[**2**].

Normal prostate epithelial cell secretions are anti-angiogenic due to the inhibitory effects of thrombospondin-1 (TSP-1), whereas this inhibitor is decreased in the pro-angiogenic secretions derived from benign prostatic hyperplasia (BPH)[**3**]. This pro-angiogenic activity depends primarily on Fibroblast Growth Factor (FGF-2) and/or VEGF, the secretion of which is increased in BPH. During disease progression in the prostate, the production of the major inhibitor TSP-1 is down-regulated, whereas that of stimulatory FGF-2 and/or VEGF is increased, leading to the induction of new vessels[**4**].

Immunolocalization studies have confirmed that the changes detected in vitro also occur in vivo. The localization of immunohistochemical staining, combined with published reports on VEGF precursor messenger ribonucleic acid (mRNA)[**5**], support the hypothesis that VEGF is synthesized predominantly by prostatic hyperplastic epithelial cells. The majority of the staining for endothelial cells could be accounted for by the VEGF’s binding to specific endothelial cell receptors. Stromal VEGF immunoreactivity could be attributed to the binding of VEGF, which is a heparin-binding growth factor, to extracellular matrix proteins[**6**] or to the production of VEGF by stromal cells. The widespread distribution of the VEGF receptor Flt-1 in BPH specimens suggests that the VEGF function in the prostate is not restricted to endothelial cells and angiogenesis[**7**].

Consistent with most reports, there is no significant VEGF expression in the normal prostatic epithelium[**7**]. Interestingly, androgens seem to regulate the VEGF expression in the prostate because castration acts through the VEGF system to inhibit angiogenesis and thereby induce apoptosis [**8**,**9**]. 

A review of literature revealed a lack of published data from the clinical studies on the therapeutic effects of anti-VEGF therapy on BPH. Thus, the evidence so far is based only on in vitro studies. Our prospective scientific experiment is a preliminary in vivo attempt to identify a potential link between anti-VEGF therapy and BPH and has revealed promising results. Beginning with our clinical observations, we initiated an experiment based on an exploratory approach. The 14 patients involved in the trial were evaluated based on both objective and subjective criteria.Uroflowmetry and International Prostate Symptom Score (I-PSS) were assessed. The main objective was to determine the potential role of intravitreal anti-VEGF therapy in improving symptoms of BPH.

## Methods

The current study was based on an exploratory trial that intended to establish whether the treatment with intravitreal Bevacizumab for wet Age-Related Macular Degeneration (AMD) had a positive effect on patients suffering from BPH, as pre-clinical studies have suggested. 

The experiment was performed between 01.08.2013 and 01.02.2014 and involved a core team of doctors from “Prof. Dr. Theodor Burghele” Hospital and the Clinical Hospital of Eye Emergencies, which were the medical units that approved the trial; both being located in Bucharest. Initially, the patients were selected in the Vitreo-Retinal Department of the Clinical Hospital of Eye Emergencies where they were diagnosed with wet AMD. They were then referred to “Prof. Dr. Theodor Burghele” Hospital for a complete urological evaluation. The Vitreo-Retinal surgeons administered the first injection of intravitreal Bevacizumab the day following the urological examination. Thereafter, patients included in the trial were monitored and evaluated by specialists from both hospitals. 

**Cohort study**

Our exploratory trial consisted of following the urological indicators of a group of 17 individuals diagnosed with BPH over the previous year, who freely consented to participate in the experiment. Of the initial 17 male subjects, 3 were excluded due to high prostate-specific antigen (PSA) values or a suspicious DRE (digital rectal examination) pending prostate biopsy. Ten of the remaining subjects were already on alpha-blocker therapy and/or phytotherapeutic agents for more than 3 months prior to the study and continued taking their medications during the investigation period. The age of the subjects in the study group ranged between 59 and 69 years, with a mean age of 64 years. The majority of the patients included in the trial did not have other systemic conditions that would interfere with the results. Two subjects had a record of mild cardiovascular disease and three of type 2, non-insulin dependent diabetes. Out of the 10 patients who were already undergoing treatment for BPH, 6 were treated with alpha-blockers and 4 were on homeopathic therapy (see **Table 1**).

**Selection criteria**

**Inclusion criteria**

Men diagnosed with wet AMD during an ophthalmological evaluation for which no previous therapy had been administered with previously diagnosed BPH, normal age-normalized PSA values, unsuspicious DRE for prostate cancer, and the ability to comply with protocol procedures, were included in the study. A written informed consent was obtained before beginning any investigational procedures.

**Exclusion criteria**

The exclusion criteria included previous anti-VEGF treatment for wet AMD, previous prostate surgery, prior Acute Urinary Retention (AUR), and concurrent therapy that alteredthe urinary flow, including cyclooxygenase 2 (COX2) inhibitors, amphetamines, anticholinergic drugs, opioids, and antidepressants. Additionally, three patients were excluded due to high initial PSA values or a suspicious DRE pending immediate prostate biopsy.

**Data collection**

The subjects were selected from a group of patients diagnosed with wet AMD for whom no prior treatment was administered. Patients were selected to undergo intravitreal anti-VEGF therapy with Avastin®. Bevacizumab (Avastin®, Roche) is a VEGF inhibitor that hinders the formation of new blood vessels. It is licensed only for intravenous administration for a number of different types of cancer at doses typically in excess of 200 mg per infusion. Bevacizumab is also administered intravitreally at a dose of 1.25 mg (0.05 ml) as an off-label treatment for a wide range of ocular diseases, such as AMD [**10**,**11**]. Anti-VEGF therapy has two primary effects: anti-angiogenic and anti-permeable (via the inhibition of choroidal neovessels and the enhancement of extravascular fluid resorption, respectively) [**12**,**13**].All the 14 subjects from the final group underwent a complete urological evaluation after signing the informed consent, which included submitting an I-PSS questionnaire and undergoing uroflowmetry the day prior to their first Bevacizumab (Avastin 0.05 ml) injection. Subjects were also followed up 30 days later, on the same day as the administration of their second Bevacizumab injection.

Factors that could have interfered with the results, such as additional treatment for BPH-related symptoms, were strictly monitored throughout the duration of the study. 

**Data analysis**

The data collection and analysis focused on one primary variable and several secondary variables[**14**]. We selected Qmax (maximum flow rate) as the primary variable, as it is capable of providing the most clinically relevant and convincing evidence directly related to the primary objective of the trial. 

Secondary variables included the following: straining, weak stream, urgency, intermittency, frequency of and incomplete emptying (I-PSS score) and quality of life (QoL) due to urinary symptoms. These indicators represented a subjective evaluation of the improvement in the urinary symptoms, as assessed by the individuals participating in the study. 

The data were analyzed by using IBM SPSS software v. 22. The significance of the differences in Qmax before and after the treatment was investigated by using a Paired Sample t-Test.

## Results

**Fig. 1:**
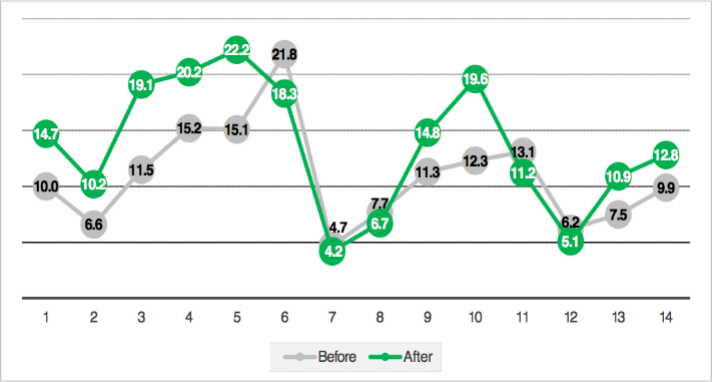
Comparison of Qmax before and after Bevacizumab administration (increases presented in absolute values)

The analysis of the primary indicator revealed a statistically significant enhancement in Qmax by an average of 2.6504 ml/sec (see **Table 2**). For the whole sample, an average increase in Qmax was registered in 9 patients, whereas 5 patients had decreased Qmax values (see **Fig. 1**).

**Table 1:** Comparison of Qmax before and after Bevacizumab administration

Patient No.	Qmax before Bevacizumab (ml/ sec)	Qmax after Bevacizumab (ml/ sec)
1	10.0	14.7
2	6.6	10.2
3	11.5	19.1
4	15.2	20.2
5	15.1	22.2
6	21.8	18.3
7	4.7	4.2
8	7.7	6.7
9	11.3	14.8
10	12.3	19.6
11	13.1	11.2
12	6.2	5.1
13	7.5	10.9
14	9.9	12.8

**Table 2:** Comparison of results before and after the administration of Bevacizumab

Patient No.	Qmax before Bevacizumab (ml/ sec)	Qmax after Bevacizumab (ml/ sec)
1	10.0	14.7
2	6.6	10.2
3	11.5	19.1
4	15.2	20.2
5	15.1	22.2
6	21.8	18.3
7	4.7	4.2
8	7.7	6.7
9	11.3	14.8
10	12.3	19.6
11	13.1	11.2
12	6.2	5.1
13	7.5	10.9
14	9.9	12.8

In the group that registered an improvement in Qmax, the average values increased by 5.05 ml/sec, from 11.175 ml/sec to 16.225 ml/sec. These results were statistically significant (p=0.000; 95% CI 3.580-6.442).

Moreover, in the group that showed decreases in Qmax, the average values decreased by 1.6 ml/sec, from 10.7 ml/sec to 9.1 ml/sec. These results were statistically significant (p=0.038; 95% CI 141-3.059).

**Fig. 2:**
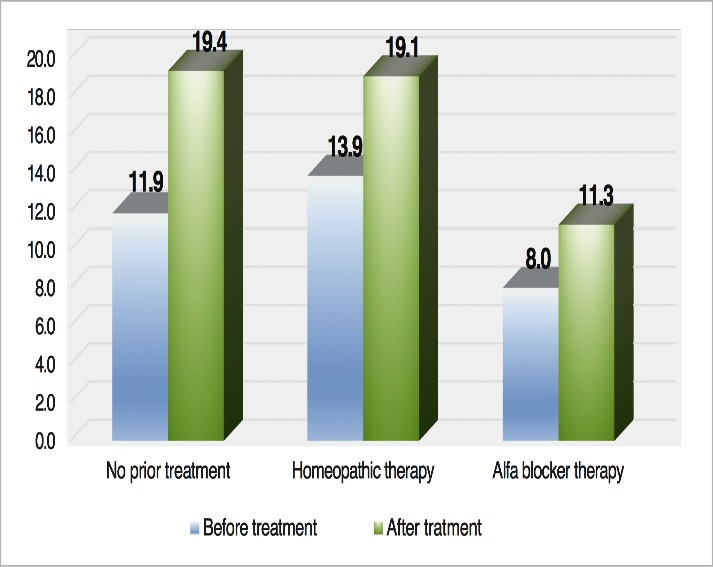
Comparison of the changes in Qmax before and after Bevacizumab administration according to prior treatment for BPH

Of the nine patients who exhibited an increase in the urinary flow, 3 were taking phytotherapeutic agents, 4 were being treated with alpha-blocker therapy, and 2 were not undergoing concurrent treatment(see **Fig. 2**).

The primary variable analyzed offered an objective approach for the study. The secondary variables presented a subjective perception of the individuals included in the study on the symptoms related to BPH. 

**Fig. 3:**
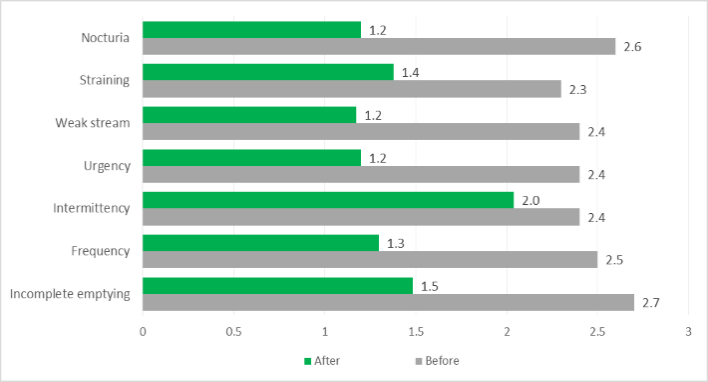
Comparison of the I-PSS indicator results before and after Bevacizumab administration (expressed as group averages)

The analysis of the secondary variables (indicators from the I-PSS score) revealed an improvement of at least 40% in each symptom per total analyzed group. The average values per indicator were considered for the analysis, as calculated at the group level (all 14 patients) on a scale from 0 to 5 (see **Fig. 3**).

Moreover, there was an overall improvement in all of the measured indicators per analyzed group: straining, weak stream, urgency, intermittency, frequency and incomplete emptying. The most notable decrease was registered in nocturia, which was defined as at least 2 voids per night. The least favorable responses were recorded for incomplete emptying, intermittency and straining.

Regarding nocturia, as assessed by Question 7 of the I-PSS questionnaire, 6 of the 14 subjects reported a decrease from baseline of more than 2 points. The same indicator, per total group, registered an overall improvement of 1.4 points. 

We calculated the average I-PSS score [**15**] per group before and after the treatment with Bevacizumab. There was an overall decrease of 1.18 points at follow-up from the initial score (mean initial score = 2.42; mean follow-up score = 1.24). 

Additionally, the indicator regarding QoL due to urinary symptoms (Question 8) was positively assessed by the majority of respondents (10 out of 14 patients) at the second injection. The average score per group improved by 1.2 points at follow-up. 

None of the patients included in our trial suffered from significant side-effects after the administration of anti-VEGF therapy [**16**].

## Comment

The exploratory trial we conducted analyzed the link between intravitreal anti-VEGF therapy for wet AMD and BPH. According to our results, intravitreal anti-VEGF injections for wet AMD had a positive effect on the BPH-related symptoms, as shown by the uroflowmetry results and the subjective evaluations of the patients (I-PSS). Both the primary and secondary variables showed improved scores at follow-up. Qmax improved by a mean of 5.05 ml/sec in 9 patients; the remaining 5 patients having a slight decrease in Qmax (mean 1.6 ml/sec). The I-PSS score improved, with an overall decrease of 1.18 points at follow-up from the initial score (mean initial score = 2.42; mean follow-up score = 1.24).

Qmax is considered one of the best measurements for diagnosing and monitoring BPH[**17**]. A Qmax value of over 15 ml/sec is usually considered normal, while a Qmax below 7 ml/sec is generally accepted as low. Of the patients included in the trial, 3 registered low Qmax values. After administrating the treatment, 2 of the patients with a low initial Qmax value did not respond to the treatment as expected, possibly due to the advanced disease. 

We used the I-PSS score to standardize the BPH-related symptoms that the patients complained about. It was intended as a complementary tool to gather additional information to that provided by Qmax. Its value consisted in providing subjective inputs from patients on the various aspects of the condition, making them more aware of their symptoms and their evolution with therapy. 

Patients reported improvements in all I-PSS indicators. We focused on nocturia as this symptom was one of the most disturbing and frequently reported by patients with BPH. In addition, this indicator is sensitive and easily quantifiable by the patients compared with the others included in the I-PSS score[**18**]. Therefore, the positive change registered in nocturia after Bevacizumab treatment was considered highly relevant in the context of this study. Patients who reported the highest decrease in the number of voids per night consistently reported the largest improvements in the QoL indicator. 

Overall, the benefits of Bevacizumab injections were greatest in patients who were not taking any medication for BPH, followed by patients on homeopathic therapies alone. Interestingly, Bevacizumab had a slight positive effect in the 4 patients already on alpha-blocker therapy for several years prior to our study. However, this might suggest that even patients on alpha-blocker therapy may benefit from concurrent anti-VEGF therapy for wet AMD. 

Medical conditions such as diabetes or cardiovascular diseases did not show any significant influence upon the outcomes of the study[**19**]. However, the associated systemic conditions were mild and well compensated through medical therapy alone. 

## Limitations

All the work for the experiment was voluntarily performed by the core team of medical professionals. Thus, no funds were obtained for the investigation, tests or related procedures. Consequently, this study included only a small number of subjects followed for a short period of time. 

Additionally, we were only able to perform a limited array of investigations (uroflowmetry, International Prostate Symptom Score (I-PSS) questionnaire) (**Table 3**) and therefore could not address the issue of Avastin bioavailability, as serum levels of Bevacizumab were not evaluated[**20**].

**Table 3:** International prostate symptom score (I-PSS)

Name:	Date:						
	Not at all	Less than 1 time in 5	Less than half the time	About half the time	More than half the time	Almost always	Your score
Incomplete emptying How often did you have a sensation of not emptying your bladder completely after you finished urinating, over the past month?	0	1	2	3	4	5	
Frequency How often did you have to urinate again less than two hours after you finished urinating, over the past month?	0	1	2	3	4	5	
Intermittency How often did you find you stopped and started again several times when you urinated, over the past month?	0	1	2	3	4	5	
Urgency How difficult did you find it to postpone urination, over the last month?	0	1	2	3	4	5	
Weak stream How often did you have a weak urinary stream, over the past month?	0	1	2	3	4	5	
Straining How often did you have to push or strain to begin urination, over the past month?	0	1	2	3	4	5	
Name:	Date:						
	None	1 time	2 times	3 times	4 times	5 times or more	Your score
Nocturia How many times did you most typically get up to urinate from the time you went to bed until the time you got up in the morning, over the past month?	0	1	2	3	4	5	
Total IPSS score							
Name:	Date:						
Quality of life due to urinary symptoms	Delighted	Pleased	Mostly satisfied	Mixed – about equally satisfied and dissatisfied	Mostly dissatisfied	Unhappy	Terrible
How would you feel about it if you were to spend the rest of your life with your urinary condition the way it is now?	0	1	2	3	4	5	6

## Conclusions

Identifying the angiogenic factors involved in prostate growth and understanding their regulation may yield useful prognostic markers that can lead to the development of anti-angiogenic strategies and therapies. However, understanding the direct effects of Bevacizumab on BPH remains challenging for specialists in the field, as there is no mention of clinical research in this area. A review of literature showed that the data regarding the therapeutic effects of anti-VEGF therapy on BPH is only based on in vitro experiments [**2**-**7**].

Whether anti-VEGF therapy could represent a future therapy for BPH, the exact mechanism of its action on the hypertrophied prostate gland has yet to be established. None of the professionals involved in this exploratory trial expected the results to be representative of all future users. We acknowledge that the methodology used for this experimental trial was limited and subjected to bias; further research must be apply a more rigorous methodological approach. So far, the results could be questioned due to the lack of control group for comparison. Additionally, although the results were statistically significant, the data should be interpreted with caution due to the very low number of subjects. 

This type of trial cannot act as the basis of a formal proof of efficacy, but it may contribute to the total body of relevant evidence. We believe that a confirmatory trial that can provide a firm support for the efficacy and safety of anti-VEGF therapy for BPH-related symptoms is mandatory before any definite conclusions are made.

**Conflicts of interest**

The authors confirm that there are no conflicts of interest. 
